# TLR4 and MMP2 polymorphisms and their associations with cardiovascular risk factors in susceptibility to aortic aneurysmal diseases

**DOI:** 10.1042/BSR20181591

**Published:** 2019-01-08

**Authors:** Tan Li, Jingjing Jing, Liping Sun, Bo Jiang, Shijie Xin, Jun Yang, Yuan Yuan

**Affiliations:** 1 Tumor Etiology and Screening Department of Cancer Institute and General Surgery, the First Hospital of China Medical University, and Key Laboratory of Cancer Etiology and Prevention (China Medical University), Liaoning Provincial Education Department, Shenyang 110001, China; 2 Department of Cardiovascular Ultrasound, the First Hospital of China Medical University, Shenyang 110001, China; 3 Department of Vascular and Thyroid Surgery, the First Hospital of China Medical University, Shenyang 110001, China

**Keywords:** aortic aneurysm, matrix metalloproteinase 2, polymorphism, toll-like receptor 4

## Abstract

**Background:** Toll-like receptor 4 (TLR4) and matrix metalloproteinase 2 (MMP2) play important roles in aortic pathophysiology. We aimed to evaluate the contribution of TLR4 and MMP2 polymorphisms individually and complex interactions between gene and risk factors in susceptibility to aortic aneurysm (AA) and its subtypes. **Methods:** KASP method was adopted to detect TLR4rs11536889, rs1927914 and MMP2rs2285053 polymorphisms in 498 controls and 472 AA patients, including 212 abdominal AA (AAA) and 216 thoracic AA (TAA). **Results:** In the overall analysis, MMP2rs2285053 TC genotype was correlated with TAA risk (*P* = 0.047, OR = 1.487). Stratified analysis revealed an increased AA risk in males with TLR4rs1927914 TC genotype, while MMP2rs2285053 TC conferred an elevated AA risk in the subjects ≤60 years, and its TC genotype and dominant model were associated with TAA in the subjects ≤60 year. The interaction between TLR4rs1927914 and MMP2rs2285053 was associated with AAA risk (*P*_interaction_ = 0.028, OR = 2.913). Furthermore, significant interaction between TLR4rs11536889 and dyslipidemia was observed for TAA risk, while TLR4rs1927914 could interact with hypertension and diabetes to increase the risk of AA or its subtypes. Two-way interaction effect of TLR4rs1927914 and MMP2rs2285053 was enhanced by diabetes or dyslipidemia. **Conclusion:** TLR4 and MMP2 polymorphisms and their complex interactions with cardiovascular risk factors contributed to aortic aneurysmal diseases.

## Introduction

Aortic aneurysm (AA) is a complex multifactorial disease characterized by structural deterioration of the vascular wall resulting in progressive dilatation and even rupture of the aorta [1]. AA can be divided into abdominal AA (AAA) and thoracic AA (TAA). Similar to other forms of cardiovascular disease, genetic variation and environmental factor accumulation modify the risk of AA formation and provide mechanistic insight into the pathogenesis of AA. AAA is classically associated with male gender, older age, hypertension and dyslipidemia [2]. TAA, in addition to conventional risk factors, exhibits a strong heritable pattern [3]. Existing evidences show that AAA and TAA share similarities in pathological states and histological phenotypes, including inflammatory reaction and extracellular matrix (ECM) degeneration in aortic wall [4,5].

Toll-like receptor 4 (TLR4) is one of the well-characterized inflammation-related molecules and plays an active role in mediating vascular inflammation and remodeling [6,7]. Its functional importance has emerged in maintaining physiological aortic homeostasis and evoking pathological aortic phenotype changes [8]. Matrix metalloproteinase 2 (MMP2) is an enzyme with proteolytic activity in degrading multiple components and has been strongly related to excessive ECM degradation in aneurysmal aorta [9–11]. Therefore, MMP2 has been widely considered a critical factor in AA initiation and development [12]. As the most common form of genetic variation, single nucleotide polymorphisms (SNPs), especially in the potential functional regions, can modulate the gene activity and function, and thus regulate the susceptibility to various disorders [13]. However, there are lacking researches on the relationship between TLR4 polymorphisms and AA risk. Although several studies have focused on the association between MMP2 polymorphisms and the risk of TAA or AAA, the results are inconsistent. There also exist many factors linked to heterogeneity, and it is difficult to recognize the mechanism of a disease from a single risk factor study. How SNP–SNP interactions and genetic associations with risk factors contributing to aortic aneurysmal diseases are poorly understood and may play a key role in the future understanding of aneurysms.

In the present study, we intended to investigate the potential effects of genetic polymorphisms in TLR4 and MMP2 individually and complex interactions in susceptibility to aortic aneurysmal diseases in a Chinese Han population. We further assessed whether above effects were modified by hypertension, diabetes and dyslipidemia in aortic aneurysmal diseases.

## Materials and methods

### Study population

The study was approved by the Ethics Committee of the First Hospital of China Medical University (Shenyang, China). Written informed consent was obtained from each participant. A total of 472 AA patients (including 212 AAA patients and 216 TAA patients) and 498 controls were involved in our study. All enrolled participants were recruited from the First Hospital of China Medical University between 2016 September and 2017 November. The diagnosis of all patients was based on the computed tomography angiography (CTA). Exclusion criteria included the subjects with coronary heart diseases, congenital heart diseases, severe vascular stenosis, autoimmune diseases, severe organ failure, infectious diseases, hematological system diseases and malignant tumors. A 5-ml fasting venous blood sample was taken from each subject for DNA isolation.

### Data collection

The demographic data and clinical related information were collected from participants’ medical records. Hypertension was defined as having a systolic blood pressure (SBP) ≥ 140 mmHg and/or having a diastolic blood pressure (DBP) ≥90 mmHg and/or being under antihypertensive treatment. Diabetes was defined as fasting serum glucose (FPG) ≥7 mmol/l (126 mg/dl) and/or being on treatment for diabetes. Dyslipidemia was defined as serum total cholesterol (TC) ≥6.22 mmol/l (240 mg/dl), or triglyceride (TG) ≥ 2.26 mmol/l (200 mg/dl), or high-density lipoprotein cholesterol (HDL-C) < 1.03 mmol/l (40 mg/dl), or low-density lipoprotein cholesterol (LDL-C) ≥4.14 mmol/l (160 mg/dl) and/or under taking hypolipidemic drugs [14].

### SNP selection and genotyping assay

A two-step approach was performed to identify tag-SNPs in TLR4 [15]. First, tag-SNPs were selected in the combinations provided by the HapMap database (http://www.HapMap.org) and Haploview software 4.2 (http://www.broadinstitute.org/mpg/haploview). Then, FastSNP Search (http://FastSNP.ibms.sinica.edu.tw/) was used to predict their potential functional effects. Accordingly, rs11536889 in the 3′-untranslated region (3′-UTR) and rs1927914 in the promoter region of TLR4 were preferred. SNP rs2285053 in the promoter region of MMP2 was chosen based on its functional importance and published data indicating its association with several cardiovascular diseases [16–18].

Genomic DNA was extracted from each blood clot using a routine phenol–chloroform method and then diluted to a working concentration of 50 ng/μl for genotyping. All samples were placed randomly on the 384-well plates and blinded for disease status. SNP genotyping was performed by Baygene Biotechnology Company Limited (Shanghai, China) using the KASP method with SNPLine platform (LGC, United Kingdom). In addition, we randomly selected 10% of the samples for repeated detection and the results were 100% consistent.

### Statistical analysis

We firstly evaluated Hardy–Weinberg equilibrium (HWE) for studied SNPs in the control group using chi-square (χ^2^) test. The distribution of baseline characteristics between case and control groups was compared by ANOVA or χ^2^ test as appropriate. The association of SNPs with aortic aneurysmal diseases risk was estimated by calculating odds ratios (ORs) and their 95% confidence intervals (95%CIs) using multivariate logistic regression after adjusting age, gender, hypertension, diabetes and dyslipidemia. The log-likelihood ratio test was performed to evaluate SNP–SNP interaction and the interaction between each SNP and potential risk factors by comparing the model only involving the main effects of each factor with the full model also containing interaction items. All the statistical analyses were carried out with SPSS 17.0 software (SPSS Inc., Chicago, IL, United States). A two-sided *P* < 0.05 was considered statistically significant. Additionally, the present study defined the dominant and recessive genetic models as heterozygote+homozygote variant vs. homozygote wild and homozygote variant vs. heterozygote+homozygote wild, respectively.

## Results

### Baseline characteristics of the study population

Baseline characteristics of the study subjects are summarized in Table 1. The present study included a total of 970 participants. There were no statistical differences in the distribution of age and gender between overall AA and control groups.

**Table 1:** Baseline characteristics of the study subjects

Variable	Controls	AA	AAA	TAA
	*n* = 498	*n* = 472	*n* = 212	*n* = 216
Age, years	60.6 ± 12.6	61.0 ± 12.6	64.9 ± 11.4^*^	58.2 ± 12.7^*^
Gender				
Male, *n* (%)	355 (71.3%)	342 (72.5%)	157 (74.1%)	149 (69.0%)
Female, *n* (%)	143 (28.7%)	130 (27.5%)	55 (25.9%)	67 (31.0%)
Hypertension				
Yes, *n* (%)	214 (43.0%)	327 (69.3%)^*^	139 (65.6%)^*^	153 (70.8%)^*^
No, *n* (%)	282 (56.6%)	125 (26.5%)	66 (31.1%)	52 (24.1%)
Missing, *n* (%)	2 (0.4%)	20 (4.2%)	7 (3.3%)	11 (5.1%)
Diabetes				
Yes, *n* (%)	55 (11.0%)	125 (26.5%)^*^	41 (19.3%)^*^	68 (31.5%)^*^
No, *n* (%)	442 (88.8%)	312 (66.1%)	161 (75.9%)	127 (58.8%)
Missing, *n* (%)	1 (0.2%)	35 (7.4%)	10 (4.7%)	21 (9.7%)
Dyslipidemia				
Yes, *n* (%)	203 (40.8%)	262 (55.5%)^*^	152 (71.7%)^*^	88 (40.7%)
No, *n* (%)	293 (58.8%)	180 (38.1%)	53 (25.0%)	106 (49.1%)
Missing, *n* (%)	2 (0.4%)	30 (6.4%)	7 (3.3%)	22 (10.2%)

* *P* vs. controls.

### Association of TLR4 and MMP2 polymorphisms with AA risk

The distribution of genotypes of TLR4rs11536889, rs1927914 and MMP2rs2285053 in each group was presented in Table 2. The genotypes in the controls were all in agreement with HWE (*P* > 0.05). First, we examined the association of each SNP with AA and its subtypes risk in the total population. After adjusting age, gender, hypertension, diabetes and dyslipidemia, only MMP2rs2285053 TC genotype was associated with an increased risk of TAA with corresponding OR of 1.487 (*P* = 0.047) (Table 2). The overall genetic effects for TLR4rs11536889 and rs1927914 related to AA and its subtypes were not found.

**Table 2:** The association between the TLR4, MMP2 polymorphisms and aortic aneurysmal diseases risk^*^

Genotypes	Controls	AA	AAA	TAA	AA vs. CON		AAA vs. CON		TAA vs. CON	
					*P*	OR (95%CI)	*P*	OR (95%CI)	*P*	OR (95%CI)
**rs11536889**										
GG	324	310	143	139						
GC	146	127	58	53	0.660	1.074 (0.783–1.473)	0.856	0.963 (0.645–1.439)	0.692	1.090 (0.712–1.668)
CC	25	29	10	19	0.366	1.340 (0.711–2.525)	0.440	1.397 (0.598–3.262)	0.136	1.771 (0.835–3.754)
GC+CC vs. GG					0.475	1.115 (0.827–1.503)	0.948	1.013 (0.692–1.482)	0.358	1.203 (0.811–1.785)
CC vs. GC+GG					0.429	1.281 (0.693–2.369)	0.460	1.369 (0.595–3.143)	0.142	1.720 (0.834–3.548)
HWE	*P* = 0.113									
**rs1927914**										
TT	190	165	75	77						
TC	214	219	99	98	0.593	1.089 (0.797–1.488)	0.671	1.090 (0.733–1.622)	0.813	1.051 (0.695–1.589)
CC	80	76	34	34	0.915	1.024 (0.662–1.585)	0.772	1.083 (0.630–1.864)	0.768	1.091 (0.611–1.950)
TC+CC vs. TT					0.677	1.065 (0.791–1.436)	0.701	1.077 (0.738–1.571)	0.756	1.064 (0.718–1.578)
CC vs. TC+TT					0.893	0.973 (0.659–1.438)	0.950	1.016 (0.620–1.665)	0.806	1.066 (0.639–1.780)
HWE	*P* = 0.137									
**rs2285053**										
CC	303	264	128	114	0.950	1.016 (0.620–1.665)	0.831	1.074 (0.556–2.075)	0.551	1.271 (0.578–2.796)
TC	167	179	70	91	0.204	1.215 (0.900–1.640)	0.959	1.010 (0.685–1.489)	**0.047**	**1.487 (1.004–2.201)**
TT	24	20	11	5	0.705	1.137 (0.585–2.211)	0.539	1.287 (0.575–2.885)	0.831	0.894 (0.321–2.493)
TC+TT vs. CC					0.199	1.208 (0.905–1.613)	0.829	1.042 (0.719–1.508)	0.072	1.420 (0.969–2.080)
TT vs. TC+CC					0.831	1.074 (0.556–2.075)	0.551	1.271 (0.578–2.796)	0.637	0.782 (0.282–2.170)
HWE	*P* = 0.873									

* *P* for association was adjusted by age, gender, hypertension, diabetes and dyslipidemia.

The results are in bold if *P  < 0.05.*

To evaluate the relationship between SNPs and aortic aneurysmal diseases in specific subgroups, we further carried out stratified analyses based on gender and age, as shown in Table 3. For rs1927914, TC genotype was only associated with an increased overall AA risk in male subjects (*P* = 0.033, OR = 1.435). For rs2285053, the heterozygote TC conferred an increased risk of AA in the subjects ≤ 60 years (*P* = 0.045, OR = 1.516); its TC genotype and dominant model were significantly correlated with elevated TAA risk in the subjects ≤ 60 years (all *P * < 0.05). As for rs11536889, no statistical significant difference between its polymorphisms and aortic aneurysmal diseases risk was observed.

**Table 3:** Association of TLR4 and MMP2 polymorphisms with the risk of aortic aneurysmal diseases stratified by age and gender

Variable	Genotypes	Controls	AA	AAA	TAA	AA vs. CON		AAA vs. CON		TAA vs. CON	
						*P*	OR (95%CI)	*P*	OR (95%CI)	*P*	OR (95%CI)
**rs11536889**											
Gender^*^											
Male	GG	229	225	102	101						
	GC	105	95	48	35	0.582	0.911 (0.652–1.272)	0.943	0.985 (0.646–1.501)	0.292	0.785 (0.500–1.232)
	CC	19	18	6	12	0.899	0.958 (0.489–1.873)	0.392	0.657 (0.251–1.719)	0.307	1.489 (0.693–3.196)
	GC+CC vs. GG					0.596	0.918 (0.670–1.259)	0.736	0.933 (0.623–1.397)	0.591	0.893 (0.591–1.349)
	CC vs. GC+GG					0.962	0.984 (0.507–1.911)	0.393	0.661 (0.255–1.711)	0.224	1.596 (0.751–3.391)
Female	GG	95	85	41	40						
	GC	41	32	10	18	0.627	0.873 (0.505–1.509)	0.164	0.573 (0.261–1.256)	0.902	1.043 (0.535–2.030)
	CC	6	11	4	7	0.176	2.047 (0.725–5.779)	0.488	1.598 (0.425–6.006)	0.085	2.761 (0.871–8.753)
	GC+CC vs. GG					0.929	1.023 (0.617–1.699)	0.320	0.700 (0.347–1.414)	0.454	1.263 (0.686–2.324)
	CC vs. GC+GG					0.146	2.140 (0.767–5.971)	0.365	1.834 (0.494–6.808)	0.083	2.727 (0.877–8.473)
Age^†^											
>60 years	GG	178	180	103	66						
	GC	84	80	43	28	0.722	0.935 (0.646–1.354)	0.501	0.859 (0.551–1.338)	0.739	0.916 (0.548–1.532)
	CC	15	16	7	9	0.890	1.053 (0.505–2.196)	0.630	0.795 (0.314–2.018)	0.262	1.650 (0.688–3.955)
	GC+CC vs. GG					0.793	0.954 (0.673–1.353)	0.447	0.849 (0.558–1.293)	0.907	1.029 (0.641–1.651)
	CC vs. GC+GG					0.845	1.075 (0.521–2.220)	0.683	0.825 (0.328–2.076)	0.227	1.701 (0.719–4.024)
≤60 years	GG	146	130	40	75						
	GC	62	47	15	25	0.482	0.852 (0.545–1.332)	0.712	0.883 (0.455–1.714)	0.409	0.796 (0.463–1.369)
	CC	10	13	3	10	0.394	1.452 (0.615–3.429)	0.926	1.065 (0.279–4.073)	0.149	1.970 (0.785–4.946)
	GC+CC vs. GG					0.754	0.936 (0.617–1.419)	0.765	0.909 (0.487–1.697)	0.866	0.958 (0.586–1.567)
	CC vs. GC+GG					0.328	1.528 (0.654–3.572)	0.869	1.118 (0.297–4.210)	0.110	2.100 (0.846–5.210)
**rs1927914**											
Gender^*^											
Male	TT	140	113	53	52						
	TC	145	168	78	72	**0.033**	**1.435 (1.029–2.002)**	0.100	1.421 (0.934–2.161)	0.224	1.306 (0.849–2.010)
	CC	60	51	23	21	0.609	1.096 (0.697–1.723)	0.757	1.097 (0.608–1.980)	0.857	0.946 (0.521–1.719)
	TC+CC vs. TT					0.079	1.324 (0.968–1.809)	0.120	1.378 (0.919–2.064)	0.401	1.190 (0.792–1.789)
	CC vs. TC+TT					0.521	0.875 (0.581–1.317)	0.614	0.872 (0.512–1.486)	0.402	0.793 (0.461–1.365)
Female	TT	50	52	22	26						
	TC	69	51	21	27	0.207	0.711 (0.418–1.208)	0.302	0.692 (0.343–1.393)	0.397	0.755 (0.394–1.447)
	CC	20	25	11	13	0.609	1.202 (0.594–2.432)	0.635	1.243 (0.506–3.053)	0.591	1.262 (0.540–2.946)
	TC+CC vs. TT					0.437	0.821 (0.501–1.346)	0.566	0.828 (0.434–1.579)	0.644	0.867 (0.474–1.586)
	CC vs. TC+TT					0.265	1.443 (0.757–2.749)	0.330	1.501 (0.663–3.396)	0.336	1.459 (0.676–3.151)
Age^†^											
>60 years	TT	111	108	57	43						
	TC	117	122	67	46	0.702	1.074 (0.744–1.551)	0.607	1.123 (0.723–1.744)	0.933	0.979 (0.597–1.605)
	CC	40	43	26	15	0.725	1.095 (0.660–1.817)	0.435	1.264 (0.702–2.277)	0.867	0.943 (0.471–1.886)
	TC+CC vs. TT					0.650	1.083 (0.768–1.527)	0.473	1.162 (0.771–1.753)	0.878	0.964 (0.607–1.533)
	CC vs. TC+TT					0.774	1.071 (0.671–1.711)	0.492	1.209 (0.703–2.080)	0.908	0.963 (0.506–1.832)
≤60 years	TT	79	57	18	35						
	TC	97	97	32	53	0.150	1.384 (0.889–2.153)	0.268	1.444 (0.754–2.765)	0.374	1.268 (0.751–2.140)
	CC	40	33	8	19	0.599	1.167 (0.656–2.077)	0.806	0.891 (0.356–2.232)	0.696	1.146 (0.578–2.275)
	TC+CC vs. TT					0.196	1.316 (0.868–1.997)	0.437	1.279 (0.687–2.382)	0.422	1.224 (0.747–2.006)
	CC vs. TC+TT					0.822	0.943 (0.567–1.570)	0.415	0.710 (0.312–1.617)	0.901	0.962 (0.526–1.760)
**rs2285053**											
Gender^*^											
Male	CC	214	190	96	78						
	TC	124	128	51	61	0.318	1.175 (0.857–1.611)	0.713	0.926 (0.613–1.398)	0.176	1.321 (0.883–1.978)
	TT	15	16	7	5	0.679	1.168 (0.561–2.433)	0.851	0.913 (0.354–2.352)	0.881	0.923 (0.324–2.627)
	TC+TT vs. CC					0.296	1.176 (0.867–1.595)	0.700	0.925 (0.622–1.376)	0.220	1.279 (0.865–1.896)
	TT vs. TC+CC					0.771	1.113 (0.541–2.292)	0.901	0.943 (0.371–2.397)	0.729	0.833 (0.295–2.347)
Female	CC	89	74	32	36						
	TC	43	51	19	30	0.170	1.429 (0.858–2.381)	0.572	1.215 (0.618–2.389)	0.070	1.759 (0.956–3.236)
	TT	9	4	4	0	0.293	0.518 (0.152–1.765)	0.849	1.130 (0.321–3.972)	NA	NA
	TC+TT vs. CC					0.333	1.274 (0.780–2.079)	0.567	1.205 (0.636–2.282)	0.215	1.459 (0.803–2.652)
	TT vs. TC+CC					0.216	0.467 (0.140–1.561)	0.882	1.097 (0.321–3.745)	NA	NA
Age^†^											
>60 years	CC	167	162	92	58						
	TC	94	96	49	40	0.778	1.053 (0.737–1.505)	0.803	0.947 (0.616–1.456)	0.434	1.210 (0.751–1.950)
	TT	16	15	11	2	0.947	0.975 (0.466–2.040)	0.525	1.302 (0.577–2.941)	0.181	0.359 (0.080–1.611)
	TC+TT vs. CC					0.821	1.040 (0.740–1.463)	0.987	0.997 (0.664–1.496)	0.689	1.099 (0.691–1.749)
	TT vs. TC+CC					0.893	0.951 (0.461–1.965)	0.492	1.323 (0.595–2.942)	0.143	0.328 (0.074–1.457)
≤60 years	CC	136	102	36	56						
	TC	73	83	21	51	**0.045**	**1.516 (1.010–2.275)**	0.752	1.103 (0.599–2.032)	**0.026**	**1.718 (1.067–2.767)**
	TT	8	5	0	3	0.755	0.833 (0.264–2.625)	NA	NA	0.898	0.914 (0.234–3.576)
	TC+TT vs. CC					0.067	1.449 (0.975–2.153)	0.979	0.992 (0.541–1.818)	**0.042**	**1.619 (1.018–2.575)**
	TT vs. TC+CC					0.552	0.709 (0.228–2.205)	NA	NA	0.653	0.734 (0.191–2.825)

* *P* for association was adjusted by age, hypertension, diabetes and dyslipidemia.

† *P* for association was adjusted by gender, hypertension, diabetes and dyslipidemia.

The results are in bold if *P  < 0.05.*

### Two-way interactions between TLR4 and MMP2 polymorphisms in AA risk

We also examined the interaction effect between TLR4 and MMP2 polymorphisms on the risk of aortic aneurysmal diseases. A combined genotype including the dominant and recessive genetic models of TLR4 SNPs, and dominant model of MMP2 SNP was used for interaction analysis. Table 4 showed that the most significant interaction was between recessive genetic model of TLR4rs1927914 and dominant model of MMP2rs2285053. This interaction was associated with an increased risk of AAA (*P*_interaction_ = 0.028, OR = 2.913).

**Table 4:** Two-way interactions between TLR4 and MMP2 polymorphisms in aortic aneurysmal diseases risk*

TLR4	Genotypes	Number of participants	MMP2rs2285053	
			CC	TC+TT
**AA vs. CON**				
rs11536889	GG	No. of controls/cases	189/171	131/133
		OR (95% CI)	1.0 (ref.)	1.306 (0.934–1.826)
	GC+CC	No. of controls/cases	113/91	58/64
		OR (95% CI)	1.041 (0.726–1.492)	1.343 (0.873–2.067)
			*P*_interaction_ = 0.764, OR = 1.099 (0.595–2.029)	
	GC+GG	No. of controls/cases	286/249	180/181
		OR (95% CI)	1.0 (ref.)	1.258 (0.952–1.664)
	CC	No. of controls/cases	16/13	9/16
		OR (95% CI)	0.957 (0.435–2.106)	2.011 (0.844–4.794)
			*P*_interaction_ = 0.353, OR = 1.812 (0.517–6.346)	
rs1927914	TT	No. of controls/cases	120/100	69/63
		OR (95% CI)	1.0 (ref.)	1.208 (0.769–1.899)
	TC+CC	No. of controls/cases	173/157	118/132
		OR (95% CI)	1.059 (0.737–1.521)	1.421 (0.972–2.078)
			*P*_interaction_ = 0.403, OR = 0.769 (0.415–1.424)	
	TC+TT	No. of controls/cases	245/226	157/150
		OR (95% CI)	1.0 (ref.)	1.114 (0.824–1.507)
	CC	No. of controls/cases	48/31	30/45
		OR (95% CI)	0.741 (0.442–1.241)	1.939 (1.164–3.230)
			*P*_interaction_ = 0.131, OR = 1.849 (0.833–4.105)	
**AAA vs. CON**				
rs11536889	GG	No. of controls/cases	189/84	131/57
		OR (95% CI)	1.0 (ref.)	1.127 (0.740–1.716)
	GC+CC	No. of controls/cases	113/44	58/23
		OR (95% CI)	0.969 (0.618–1.521)	0.967 (0.547–1.710)
			*P*_interaction_ = 0.466, OR = 1.347 (0.605–3.000)	
	GC+GG	No. of controls/cases	286/123	180/75
		OR (95% CI)	1.0 (ref.)	1.075 (0.754–1.532)
	CC	No. of controls/cases	16/5	9/5
		OR (95% CI)	0.837 (0.299–2.342)	1.191 (0.359–3.949)
			*P*_interaction_ = 0.468, OR = 1.851 (0.351–9.762)	
rs1927914	TT	No. of controls/cases	120/47	69/28
		OR (95% CI)	1.0 (ref.)	1.021 (0.583–1.787)
	TC+CC	No. of controls/cases	173/77	118/53
		OR (95% CI)	1.138 (0.736–1.757)	1.115 (0.694–1.792)
			*P*_interaction_ = 0.439, OR = 0.748 (0.359–1.560)	
	TC+TT	No. of controls/cases	245/112	157/59
		OR (95% CI)	1.0 (ref.)	0.787 (0.538–1.153)
	CC	No. of controls/cases	48/12	30/22
		OR (95% CI)	0.574 (0.293–1.125)	1.705 (0.937–3.102)
			***P*_interaction_ = 0.028, OR = 2.913 (1.119–7.585)**	
**TAA vs. CON**				
rs11536889	GG	No. of controls/cases	189/73	131/62
		OR (95% CI)	1.0 (ref.)	1.490 (0.953–2.329)
	GC+CC	No. of controls/cases	113/39	58/33
		OR (95% CI)	1.136 (0.696–1.853)	1.707 (0.983–2.964)
			*P*_interaction_ = 0.896, OR = 1.055 (0.472–2.356)	
	GC+GG	No. of controls/cases	286/104	180/84
		OR (95% CI)	1.0 (ref.)	1.404 (0.968–2.036)
	CC	No. of controls/cases	16/8	9/11
		OR (95% CI)	1.331 (0.504–3.514)	3.550 (1.364–9.241)
			*P*_interaction_ = 0.285, OR = 2.254 (0.509–9.987)	
rs1927914	TT	No. of controls/cases	120/46	69/29
		OR (95% CI)	1.0 (ref.)	1.144 (0.626–2.090)
	TC+CC	No. of controls/cases	173/65	118/64
		OR (95% CI)	0.930 (0.569–1.520)	1.538 (0.940–2.518)
			*P*_interaction_ = 0.649, OR = 1.208 (0.535–2.730)	
	TC+TT	No. of controls/cases	245/97	157/73
		OR (95% CI)	1.0 (ref.)	1.256 (0.843–1.872)
	CC	No. of controls/cases	48/14	30/20
		OR (95% CI)	0.785 (0.388–1.592)	2.199 (1.166–4.148)
			*P*_interaction_ = 0.121, OR = 2.299 (0.802–6.589)	

* *P* for association was adjusted by age, gender, hypertension, diabetes and dyslipidemia.

The results are in bold if *P  *for interaction* < 0.05.*

### Interaction effects of TLR4/MMP2 SNPs with potential cardiovascular risk factors on AA risk

We investigated the interaction effects of TLR4 and MMP2 polymorphisms with potential cardiovascular risk factors, including hypertension, diabetes and dyslipidemia, in the susceptibility to aortic aneurysmal diseases, as shown in Table 5. With adjustments for age, gender, hypertension, diabetes and dyslipidemia status unless the risk factor was regarded as an interaction item, the results indicated that CC genotype of TLR4rs11536889 had a positive interaction effect with dyslipidemia on TAA risk (*P*_interaction_ = 0.001). For TLR4rs1927914 polymorphism, CC genotype was positively interactive with hypertension on the risk of overall AA and AAA (*P*_interaction_ = 0.018 and 0.039, respectively), in addition, the interactions of diabetes with CC genotype on AA and TAA risk (*P*_interaction_ = 0.032 and 0.018, respectively), and with TC+CC genotype on AAA risk (*P*_interaction_ = 0.040) were observed. However, there were no significant interactions between MMP2rs2285053 and risk factors in aortic aneurysmal diseases risk.

**Table 5:** The interaction effects between the TLR4, MMP2 polymorphisms and risk factors in the susceptibility to aortic aneurysmal diseases

SNP genotyps	Number of participants	Hypertension*		Diabetes^†^		Dyslipidemia^‡^	
		No	Yes	No	Yes	No	Yes
**AA vs. CON**							
rs11536889							
GG	No. of controls/cases	177/77	145/221	289/202	34/85	190/107	132/177
	OR (95% CI)	1.0 (ref.)	3.367 (2.360–4.804)	1.0 (ref.)	3.410 (2.170–5.358)	1.0 (ref.)	2.224 (1.589–3.113)
GC+CC	No. of controls/cases	102/47	69/101	150/106	21/38	102/70	69/83
	OR (95% CI)	1.171 (0.748–1.833)	3.500 (2.300–5.327)	1.063 (0.777–1.453)	2.754 (1.562–4.853)	1.198 (0.808–1.777)	2.016 (1.341–3.032)
		*P*_interaction_ = 0.851, OR = 0.944 (0.516–1.727)		*P*_interaction_ = 0.426, OR = 0.729 (0.335–1.587)		*P*_interaction_ = 0.409, OR = 0.777 (0.428–1.413)	
GC+GG	No. of controls/cases	265/118	203/302	416/288	53/117	272/167	196/241
	OR (95% CI)	1.0 (ref.)	3.172 (2.374–4.240)	1.0 (ref.)	3.064 (2.122–4.423)	1.0 (ref.)	1.899 (1.440–2.505)
CC	No. of controls/cases	14/6	11/20	23/20	2/6	20/10	5/19
	OR (95% CI)	1.049 (0.393–2.803)	4.007 (1.831–8.768)	1.211 (0.641–2.286)	4.640 (0.930–23.162)	0.779 (0.346–1.753)	5.192 (1.852–14.561)
		*P*_interaction_ = 0.604, OR = 1.407 (0.386–5.126)		*P*_interaction_ = 0.821, OR = 1.230 (0.203–7.447)		*P*_interaction_ = 0.060, OR = 3.710 (0.945–14.565)	
rs1927914							
TC+TT	No. of controls/cases	223/106	181/258	360/266	43/87	238/148	165/210
	OR (95% CI)	1.0 (ref.)	2.794 (2.052–3.806)	1.0 (ref.)	2.635 (1.746–3.975)	1.0 (ref.)	1.884 (1.399–2.537)
CC	No. of controls/cases	52/15	26/61	72/38	8/34	49/24	31/48
	OR (95% CI)	0.570 (0.296–1.094)	4.820 (2.854–8.139)	0.780 (0.507–1.199)	5.717 (2.584–12.648)	0.787 (0.456–1.360)	2.633 (1.588–4.366)
		***P*_interaction_ = 0.018, OR = 2.795 (1.193–6.548)**		***P*_interaction_ = 0.032, OR = 3.001 (1.099–8.192)**		*P*_interaction_ = 0.196, OR = 1.689 (0.763–3.736)	
TT	No. of controls/cases	108/47	82/110	168/119	21/33	113/68	76/87
	OR (95% CI)	1.0 (ref.)	3.016 (1.900–4.789)	1.0 (ref.)	2.291 (1.239–4.236)	1.0 (ref.)	1.854 (1.191–2.887)
TC+CC	No. of controls/cases	167/74	125/209	264/185	30/88	174/104	120/171
	OR (95% CI)	1.028 (0.652–1.620)	3.614 (2.365–5.523)	0.997 (0.733–1.356)	3.869 (2.379–6.292)	1.023 (0.688–1.522)	2.262 (1.527–3.350)
		*P*_interaction_ = 0.632, OR = 1.160 (0.633–2.126)		*P*_interaction_ = 0.094, OR = 1.989 (0.888–4.454)		*P*_interaction_ = 0.351, OR = 1.329 (0.731–2.415)	
rs2285053							
CC	No. of controls/cases	173/78	129/176	269/91	34/29	181/97	121/146
	OR (95% CI)	1.0 (ref.)	2.667 (1.854–3.836)	1.0 (ref.)	3.020 (1.898–4.807)	1.0 (ref.)	2.043 (1.433–2.914)
TC+TT	No. of controls/cases	105/45	85/144	169/67	21/12	110/80	80/111
	OR (95% CI)	0.928 (0.587–1.465)	3.794 (2.578–5.584)	1.284 (0.947–1.740)	4.193 (2.404–7.314)	1.343 (0.911–1.980)	2.529 (1.716–3.726)
		*P*_interaction_ = 0.124, OR = 1.600 (0.879–2.912)		*P*_interaction_ = 0.680, OR = 1.174 (0.547–2.519)		*P*_interaction_ = 0.785, OR = 0.923 (0.518–1.644)	
**AAA vs. CON**							
rs11536889							
GG	No. of controls/cases	177/44	145/95	289/106	34/30	190/31	132/105
	OR (95% CI)	1.0 (ref.)	2.488 (1.614–3.836)	1.0 (ref.)	2.253 (1.284–3.953)	1.0 (ref.)	4.592 (2.880–7.324)
GC+CC	No. of controls/cases	102/22	69/43	150/54	21/11	102/22	69/46
	OR (95% CI)	0.915 (0.516–1.623)	2.522 (1.507–4.222)	0.991 (0.672–1.463)	1.498 (0.698–3.217)	1.290 (0.703–2.369)	3.814 (2.215–6.570)
		*P*_interaction_ = 0.504, OR = 1.303 (0.600–2.831)		*P*_interaction_ = 0.277, OR = 0.564 (0.201–1.583)		*P*_interaction_ = 0.493, OR = 0.759 (0.345–1.669)	
GC+GG	No. of controls/cases	265/63	203/132	416/150	53/41	272/48	196/146
	OR (95% CI)	1.0 (ref.)	2.581 (1.801–3.700)	1.0 (ref.)	2.055 (1.294–3.263)	1.0 (ref.)	4.033 (2.752–5.911)
CC	No. of controls/cases	14/3	11/6	23/10	2/0	20/5	5/5
	OR (95% CI)	0.936 (0.261–3.359)	2.382 (0.847–6.695)	1.130 (0.511–2.499)	NA	1.467 (0.525–4.105)	4.696 (1.216–18.139)
		*P*_interaction_ = 0.764, OR = 1.298 (0.237–7.118)		*P*_interaction_ = NA, OR = NA		*P*_interaction_ = 0.946, OR = 1.062 (0.187–6.021)	
rs1927914							
TC+TT	No. of controls/cases	223/59	181/108	360/136	43/29	238/45	165/123
	OR (95% CI)	1.0 (ref.)	2.137 (1.457–3.133)	1.0 (ref.)	3.867 (1.522–9.829)	1.0 (ref.)	3.591 (2.402–5.368)
CC	No. of controls/cases	52/6	26/28	72/21	8/12	49/6	31/27
	OR (95% CI)	0.455 (0.186–1.114)	3.946 (2.128–7.319)	0.844 (0.498–1.430)	1.741 (1.026–2.955)	0.561 (0.212–1.488)	4.848 (2.630–8.935)
		***P*_interaction_= 0.039, OR = 3.256 (1.064–9.967)**		*P*_interaction_ = 0.098, OR = 2.902 (0.823–10.237)		*P*_interaction_= 0.278, OR = 1.900 (0.596–6.050)	
TT	No. of controls/cases	108/27	82/46	168/62	21/9	113/24	76/49
	OR (95% CI)	1.0 (ref.)	2.223 (1.256–3.936)	1.0 (ref.)	1.281 (0.553–2.970)	1.0 (ref.)	2.883 (1.613–5.153)
TC+CC	No. of controls/cases	167/38	125/90	264/95	30/32	174/27	120/101
	OR (95% CI)	0.939 (0.535–1.649)	2.748 (1.637–4.611)	0.978 (0.668–1.432)	2.658 (1.467–4.815)	0.732 (0.398–1.346)	3.765 (2.228–6.362)
		*P*_interaction_ = 0.452, OR = 1.342 (0.624–2.886)		***P*_interaction_ = 0.040, OR = 3.008 (1.052–9.066)**		*P*_interaction_ = 0.130, OR = 1.834 (0.837–4.022)	
rs2285053							
CC	No. of controls/cases	173/44	129/81	269/91	34/29	181/30	121/91
	OR (95% CI)	1.0 (ref.)	2.276 (1.454–3.563)	1.0 (ref.)	2.402 (1.364–4.230)	1.0 (ref.)	4.206 (2.597–6.812)
TC+TT	No. of controls/cases	105/21	85/56	169/67	21/12	110/22	80/59
	OR (95% CI)	0.855 (0.479–1.527)	2.714 (1.679–4.390)	1.215 (0.834–1.770)	1.728 (0.796–3.751)	1.196 (0.650–2.201)	4.321 (2.564–7.282)
		*P*_interaction_ = 0.457, OR = 1.338 (0.622–2.877)		*P*_interaction_ = 0.172, OR = 0.488 (0.174–1.366)		*P*_interaction_ = 0.700, OR = 0.859 (0.397–1.861)	
**TAA vs. CON**							
rs11536889							
GG	No. of controls/cases	177/29	145/104	289/80	34/44	190/65	132/56
	OR (95% CI)	1.0 (ref.)	4.100 (2.473–6.797)	1.0 (ref.)	4.647 (2.714–7.958)	1.0 (ref.)	1.059 (0.678–1.652)
GC+CC	No. of controls/cases	102/22	69/45	150/45	21/21	102/38	69/31
	OR (95% CI)	1.493 (0.791–2.816)	4.308 (2.422–7.662)	1.161 (0.754–1.787)	3.948 (2.027–7.687)	1.058 (0.654–1.710)	1.206 (0.710–2.050)
		*P*_interaction_ = 0.509, OR = 0.759 (0.335–1.721)		*P*_interaction_ = 0.516, OR = 0.733 (0.287–1.871)		*P*_interaction_ = 0.925, OR = 1.039 (0.467–2.315)	
GC+GG	No. of controls/cases	265/48	203/135	416/114	53/60	272/98	196/73
	OR (95% CI)	1.0 (ref.)	3.411 (2.287–5.086)	1.0 (ref.)	3.971 (2.550–6.184)	1.0 (ref.)	0.918 (0.633–1.332)
CC	No. of controls/cases	14/3	11/14	23/10	2/6	20/5	5/14
	OR (95% CI)	1.337 (0.368–4.850)	6.805 (2.821–16.417)	1.584 (0.712–3.528)	12.147 (2.416–61.069)	0.587 (0.196–1.762)	6.457 (2.185–19.075)
		*P*_interaction_ = 0.495, OR = 1.752 (0.349–8.780)		*P*_interaction_ = 0.460, OR = 2.066 (0.301–14.156)		***P*_interaction_ = 0.001, OR = 15.724 (2.949–83.835)**	
rs1927914							
TC+TT	No. of controls/cases	223/42	181/122	360/111	43/45	238/85	165/70
	OR (95% CI)	1.0 (ref.)	3.358 (2.186–5.157)	1.0 (ref.)	3.117 (1.895–5.127)	1.0 (ref.)	1.018 (0.685–1.513)
CC	No. of controls/cases	52/7	26/27	72/13	8/19	49/16	31/16
	OR (95% CI)	0.826 (0.348–1.961)	5.431 (2.800–10.533)	0.623 (0.325–1.196)	8.182 (3.455–19.374)	0.938 (0.498–1.766)	1.500 (0.768–2.932)
		*P*_interaction_ = 0.271, OR = 1.848 (0.619–5.517)		***P*_interaction_ = 0.018, OR = 4.278 (1.285–14.240)**		*P*_interaction_ = 0.235, OR = 1.864 (0.667–5.212)	
TT	No. of controls/cases	108/19	82/52	168/50	21/19	113/37	76/32
	OR (95% CI)	1.0 (ref.)	3.555 (1.873–6.746)	1.0 (ref.)	3.055 (1.463–6.379)	1.0 (ref.)	1.206 (0.671–2.167)
TC+CC	No. of controls/cases	167/30	125/97	264/74	30/45	174/64	120/54
	OR (95% CI)	1.071 (0.551–2.082)	4.134 (2.276–7.509)	0.976 (0.637–1.496)	4.836 (2.701–8.660)	1.197 (0.736–1.945)	1.2550.746–2.111)
		*P*_interaction_ = 0.977, OR = 1.012 (0.440–2.326)		*P*_interaction_ = 0.312, OR = 1.650 (0.625–4.355)		*P*_interaction_ = 0.709, OR = 1.165 (0.522–2.602)	
rs2285053							
CC	No. of controls/cases	173/29	29/79	269/66	34/34	181/59	121/41
	OR (95% CI)	1.0 (ref.)	2.963 (1.782–4.926)	1.0 (ref.)	3.727 (2.099–6.618)	1.0 (ref.)	0.866 (0.529–1.417)
TC+TT	No. of controls/cases	105/22	85/69	169/57	21/32	110/45	80/44
	OR (95% CI)	1.113 (0.584–2.121)	4.647 (2.759–7.826)	1.374 (0.901–2.094)	6.562 (3.475–12.390)	1.261 (0.791–2.011)	1.574 (0.964–2.572)
		*P*_interaction_ = 0.273, OR = 1.576 (0.699–3.556)		*P*_interaction_ = 0.455, OR = 1.414 (0.570–3.508)		*P*_interaction_ = 0.363, OR = 1.435 (0.659–3.126)	

* *P* for interaction was adjusted by age, gender, diabetes and dyslipidemia.

^†^, *P* for interaction was adjusted by age, gender, hypertension and dyslipidemia.

^‡^, *P* for interaction was adjusted by age, gender, hypertension and diabetes.

The results are in bold if *P  *for interaction* < 0.05.*

Further, we tested the influence of hypertension, diabetes and hyperlipidemia on the interaction strength between TLR4rs1927914 and MMP2rs2285053 (Table 6). Under conditions of diabetes and dyslipidemia, the interaction effect on AAA risk was significantly enhanced with corresponding ORs of 22.905 (*P*_interaction_ = 0.013) and 3.702 (*P*_interaction_ = 0.042), respectively. Interestingly, TLR4rs1927914 conferred a significant positive interaction with MMP2rs2285053 for AA risk in the condition of diabetes (*P*_interaction_ = 0.038, OR = 8.507). However, hypertension did not influence TLR4rs1927914–MMP2rs2285053 interaction effect for aortic aneurysmal diseases.

**Table 6:** The effect of potential risk factors on the interaction between TLR4rs1927914 and MMP2rs2285053 polymorphisms in aortic aneurysmal diseases risk

TLR4	MMP2	AA vs. CON				AAA vs. CON				TAA vs. CON			
rs1927914	rs2285053	No. of controls/cases	OR (95%CI)	No. of controls/cases	OR (95%CI)	No. of controls/cases	OR (95%CI)	No. of controls/cases	OR (95%CI)	No. of controls/cases	OR (95%CI)	No. of controls/cases	OR (95%CI)
			Hypertension (+)*		Hypertension (−)*		Hypertension (+)*		Hypertension (−)*		Hypertension (+)^*^		Hypertension (−)*
TC+TT	CC	112/147	1 (ref)	133/69	1 (ref)	112/70	1 (ref)	133/39	1 (ref)	112/65	1 (ref)	133/26	1 (ref)
TC+TT	TC+TT	69/105	1.337 (0.894–1.998)	88/35	0.749 (0.451–1.244)	69/36	0.974 (0.584–1.623)	88/19	0.816 (0.440–1.515)	69/53	1.557 (0.944–2.567)	88/15	0.709 (0.330–1.521)
CC	CC	13/24	1.448 (0.688–3.048)	34/7	0.367 (0.146–0.918)	13/8	1.005 (0.381–2.654)	34/4	0.435 (0.145–1.305)	13/12	1.582 (0.635–3.946)	34/2	0.326 (0.073–1.448)
CC	TC+TT	13/37	2.534 (1.275–6.036)	16/8	0.909 (0.356–2.320)	13/20	2.728 (1.261–5.904)	16/2	0.462 (0.101–2.102)	13/15	2.462 (1.077–5.624)	16/5	1.732 (0.580–5.173)
			*P*_interaction_= 0.590, OR = 1.337 (0.466–3.836)		*P*_interaction_= 0.144, OR = 2.977 (0.689–12.862)		*P*_interaction_= 0.187, OR = 2.422 (0.650–9.025)		*P*_interaction_= 0.693, OR = 1.506 (0.196–11.551)		*P*_interaction_= 0.663, OR = 1.340 (0.360–4.986)		*P*_interaction_= 0.065, OR = 6.852 (0.890–52.775)
			Diabetes (+)^†^		Diabetes (−)^†^		Diabetes (+)^†^		Diabetes (−)^†^		Diabetes (+)^†^		Diabetes (−)^b^
TC+TT	CC	26/55	1 (ref)	219/149	1 (ref)	26/24	1 (ref)	219/80	1 (ref)	26/26	1 (ref)	219/59	1 (ref)
TC+TT	TC+TT	17/30	0.934 (0.427–2.041)	139/111	1.182 (0.847–1.649)	17/5	0.295 (0.086–1.015)	139/53	1.086 (0.717–1.645)	17/17	1.219 (0.488–3.041)	139/49	1.278 (0.811–2.015)
CC	CC	6/13	1.106 (0.372–3.293)	42/15	0.550 (0.289–1.046)	6/5	0.788 (0.197–3.153)	42/7	0.505 (0.217–1.175)	6/7	1.517 (0.440–5.223)	42/5	0.403 (0.138–1.175)
CC	TC+TT	2/21	5.255 (1.134–24.361)	28/23	1.372 (0.756–2.489)	2/7	4.136 (0.778–21.993)	28/14	1.535 (0.764–3.082)	2/12	7.150 (1.421–35.967)	28/8	1.224 (0.526–2.848)
			***P*_interaction_= 0.038, OR = 8.507 (1.128–64.161)**		*P*_interaction_= 0.447, OR = 1.148 (0.804–1.639)		***P*_interaction_= 0.013, OR = 22.905 (1.954–268.517)**		*P*_interaction_= 0.466, OR = 1.183 (0.753–1.857)		*P*_interaction_= 0.097, OR = 7.564 (0.695–82.302)		*P*_interaction_= 0.394, OR = 1.229 (0.765–1.975)
			Dyslipidemia (+)^‡^		Dyslipidemia (−)^‡^		Dyslipidemia (+)^‡^		Dyslipidemia (−)^‡^		Dyslipidemia (+)^‡^		Dyslipidemia (−)^‡^
TC+TT	CC	101/126	1 (ref)	144/81	1 (ref)	101/81	1 (ref)	144/25	1 (ref)	101/34	1 (ref)	144/50	1 (ref)
TC+TT	TC+TT	64/79	1.018 (0.658–1.573)	92/65	1.249 (0.814–1.917)	64/40	0.789 (0.475–1.311)	92/19	1.253 (0.650–2.416)	64/33	1.639 (0.879–3.055)	92/34	1.044 (0.618–1.763)
CC	CC	17/17	0.833 (0.395–1.756)	31/12	0.695 (0.330–1.463)	17/8	0.660 (0.270–1.612)	31/3	0.600 (0.170–2.122)	17/6	1.143 (0.386–3.386)	31/7	0.613 (0.240–1.563)
CC	TC+TT	12/31	2.660 (1.270–5.574)	18/12	1.158 (0.520–2.577)	12/19	2.423 (1.087–5.402)	18/3	0.667 (0.145–3.059)	12/10	3.521 (1.354–9.211)	18/9	1.532 (0.645–3.639)
			*P*_interaction_ = 0.093, OR = 2.565 (0.853–7.709)		*P*_interaction_ = 0.403, OR = 1.222 (0.764–1.956)		***P*_interaction_ = 0.042, OR = 3.702 (1.048–13.080)**		*P*_interaction_ = 0.852, OR = 0.821 (0.103–6.528)		*P*_interaction_ = 0.277, OR = 2.399 (0.495–11.634)		*P*_interaction_ = 0.305, OR = 2.155 (0.498–9.327)

* *P* for interaction was adjusted by age, gender, diabetes and dyslipidemia.

† *P* for interaction was adjusted by age, gender, hypertension and dyslipidemia.

‡ *P* for interaction was adjusted by age, gender, hypertension and diabetes.

The results are in bold if *P  *for interaction* < 0.05.*

## Discussion

TLR4 and MMP2 have been considered as candidate genes which play crucial roles in the pathogenesis of AA, which can be affected by complex genetic and environmental factors. The present study focused on both gene polymorphisms and their associations with cardiovascular risk factors in susceptibility to AA as well as its subtypes. To the best of our knowledge, this is the first study to evaluate the associations of TLR4rs11536889, rs1927914 and MMP2rs2285053 polymorphisms with AA risk in a Chinese Han population.

TLR4 is located on chromosome 9q32-q33 and has been believed to link inflammation to aneurysms [19,20]. Several studies have reported a functional significance of TLR4 polymorphism. TLR4rs11536889, located in the 3′-UTR, is considered a functional SNP because of its contribution to regulating TLR4 translation by binding to microRNAs [21]. In addition, the rs1927914 SNP, located in the promoter of TLR4, can influence transcriptional factor binding site, modify the promoter activity and regulate gene expression or signaling pathway [22,23]. Recently, several studies have reported that TLR4rs11536889 and rs1927914 polymorphisms had impacts on human inflammatory and malignant diseases [24–26]. Moreover, Sun et al. demonstrated that TLR4 rs11536889 was a novel genetic factor in the development of coronary artery disease, influencing its angiographic extent and severity [27], while Xu et al. found that rs1927914 was correlated with susceptibility to diabetes and diabetic retinopathy in a Chinese Han population [24]. In our study, after adjusting the potential confounders, there were no associations of TLR4rs11536889 and rs1927914 polymorphisms with the risk of AA or its subtypes in the overall analysis. For the stratified analyses, rs1927914 TC genotype was only correlated with susceptibility to male AA subjects. Generally speaking, males are more vulnerable to AA formation than females, which may ascribe to the protective effect of estrogens on inflammatory responses [28]. Therefore, the relationship between TLR4rs1927914 polymorphisms and AA risk was eminently reflected in male subgroup.

As for MMP2, it is located on chromosome 16q13-21 and contributes to vascular protein degradation and aortic wall destruction [12]. MMP2rs2285053 is located in the promoter region and its variation has been reported to disrupt promoter activity [29]. To date, several studies have evaluated MMP2rs2285053 polymorphisms in relation to the susceptibility of cardiovascular diseases but the results are conflicting. MMP2rs2285053 polymorphisms were found to be associated with increased risks of myocardial infarction [30] and degenerative mitral valve disease [16], and lower risks of carotid atherosclerosis-vulnerable plaque [31] and chronic heart failure [32], but not related to the risks of atrial fibrillation [33] and coronary artery disease [34]. In the present study, we showed that MMP2rs2285053 TC genotype was associated with an increased TAA risk in the whole analysis. Additionally, in the stratified analyses, rs2285053 polymorphisms had increased risk effects on AA and TAA in younger subjects (age ≤ 60 years). Although the wall thickness-to-lumen size is consistent throughout the aorta, thoracic and abdominal aortas may have diverse sensitivity to different pathological stimuli [35,36]. For instance, thoracic aortic wall consists of a higher content of elastin, which can make TAA more susceptible to MMP2 polymorphisms in some conditions.

The ability in recognizing AA susceptibility for one single polymorphism locus is limited, however, when multiple SNPs are combined for detection, more advantages could be obtained [37,38]. Our results suggested that when analyzed individually, neither TLR4rs1927914 nor MMP2rs2285053 polymorphisms had an effect on AAA risk, in contrast, their combined effect was significantly related to an increased risk of AAA with an OR value of 2.913. The interaction effect of two or more genes can account for a phenomenon in the missing heritability of many diseases, which is often underestimated or even ignored. Consequently, the effects of TLR4rs1927914 and MMP2rs2285053 interaction on the pathogenesis of AAA might depend on the presence of the other SNP. Numerous experiments have demonstrated that activation of TLR4 signaling could promote MMP2 expression and secretion from a variety of cells [20,39,40]. In addition, during aortic tissue damage and remodeling process, released fragments from ECM degradation can also trigger TLR4 signaling [41]. In 2014, Ruvolo et al. found that TLR4rs4986790 polymorphism conferred a higher susceptibility for sporadic TAA and it represented, together with ACErs1799752 D, MMP9rs3918242 T and MMP2rs2285053 T alleles, an independent sporadic TAA risk factor, which indicated that TLR4 contributed to vascular homeostasis by creating a cross-talk network with other pathways [18]. These observations could in part account for the interaction effect between TLR4 and MMP2 polymorphisms in our study.

Being a multifactorial and multistep disease of AA, there might be complex interactions between the risk allele and confounding factors in a stronger combination rather than individually. Further, we performed the interaction of TLR4rs11536889, rs1927914 and MMP2rs2285053 with potential cardiovascular risk factors, including hypertension, diabetes and dyslipidemia, in the risk of aortic aneurysmal diseases. Interestingly, significant interaction with risk factors was only demonstrated in TLR4 polymorphisms. In detail, TLR4rs11536889 was interactive with dyslipidemia to increase TAA risk, while rs1927914 polymorphisms were associated with hypertension in the overall AA and AAA risk, and correlated with diabetes in the pathogenesis of AA and its subtypes. As a well-characterized pattern-recognition receptor, TLR4 can be activated by various exogenous and endogenous ligands. Hypertension, a known risk factor for AA, is regarded as a low-grade inflammatory disease and can enhance TLR4 expression and activity [42,43]. Hernanz et al. showed that TLR4 up-regulation induced by AngII contributed to the inflammation, endothelial dysfunction and vascular remodeling associated with hypertension [44]. Diabetes seems to be a protective factor for AAA, but present knowledge is far from definitive and its impact on TAA has been poorly investigated [45–47]. Several studies have found that high glucose can activate TLR4 expression and function in monocytes and human aortic endothelial cell in the process of vascular inflammation [48,49]. Recent data also demonstrated that blood lipid was able to facilitate inflammatory signaling pathways and its effects on cells can be mediated by binding to TLR4 [50,51]. Moreover, dyslipidemia contributes to an increase in several endogenous ligands for TLR4 including hyaluronic acid, biglycan and oxidized LDL [52,53]. Aortic wall is continuously under stimulation from cardiovascular risk factors, leading to a dynamic damage. Besides, above endogenous factors induced TLR4 activation and its downstream inflammation might add additional risks for AA occurrence. These evidences together may, at least in part, explain why a more perceptible association of TLR4rs11536889 and rs1927914 polymorphisms with aortic aneurysmal diseases in the presence of risk factors was observed. Furthermore, the enhanced interaction strength of TLR4rs1927914 and MMP2rs2285053 polymorphisms under diabetic or dyslipidemia status was found in the current study. Therefore, it is reasonable to hypothesize that the SNP–SNP interaction might elevate inflammatory status, making aortic wall more sensitive and vulnerable to risk factors.

There are some limitations in our study. First, the sample size was relatively small for stratification and interaction analyses, especially for rare genotypes, and thereby our results required the validation and replication in larger populations. Second, information on lifestyle factors (such as smoking and drinking) was lacking and precluded their use as potential interaction analysis with SNPs. In addition, since AA is a multifactorial disease, specific and few polymorphisms of TLR4 and MMP2 in the present study may not fully explain susceptibility to aortic aneurysmal diseases. More comprehensive studies involving multiple related gene SNPs, gene–gene and gene–environment interactions are needed in the future. Moreover, considering the clinical significance of the study, further genotype and phenotype correlation analysis will be considered to observe biological effects of reported polymorphisms in patients’ samples in terms of altered level or activity of TLR4 and MMP2.

In summary, the present study for the first time reported that TLR4rs1927914 and MMP2rs2285053 polymorphisms were linked to the susceptibility to aortic aneurysmal diseases in overall or stratified analysis in a Chinese population. A novel SNP–SNP interaction between TLR4rs1927914 and MMP2rs2285053 associated with an increased AAA risk was observed. Genetic variant of TLR4rs11536889 could interact with dyslipidemia to increase the risk of TAA, whereas TLR4rs1927914 polymorphisms had interaction effects with hypertension and diabetes in the risk of AA or its subtypes. Moreover, the interaction strength of TLR4rs1927914 and MMP2rs2285053 polymorphisms was further enhanced when combined with diabetes or dyslipidemia. Therefore, our study might offer a topic for future large-scale research and further molecular mechanism evidences are still required to verify our findings.

## Abbreviations

AA: aortic aneurysm
AAA: abdominal aortic aneurysm
CI: confidence interval
CTA: computed tomography angiography
DBP: diastolic blood pressure
ECM: extracellular matrix
FPG: fasting serum glucose
HDL-C: high-density lipoprotein cholesterol
HWE: Hardy–Weinberg equilibrium
LDL-C: low-density lipoprotein cholesterol
MMP2: matrix metalloproteinase 2
OR: odds ratio
SBP: systolic blood pressure
SNP: single nucleotide polymorphism
TAA: thoracic aortic aneurysm
TC: total cholesterol
TG: triglyceride
TLR4: toll-like receptor 4
UTR: untranslated region
